# Association between personality traits of dairy cows and their peripubertal heifer offspring

**DOI:** 10.3168/jdsc.2022-0365

**Published:** 2023-07-13

**Authors:** S.C. Czachor, A.J. Schwanke, J.E. Brasier, B.J. Van Soest, T.J. DeVries

**Affiliations:** Department of Animal Biosciences, University of Guelph, Guelph, Ontario, N1G 2W1, Canada

## Abstract

This study aimed to determine if personality traits identified in dairy cows during the transition phase are correlated with those of their peripubertal heifer calves. At ~24 d before calving and ~24 d after being first introduced to the automated milking system, the personality traits of 23 Holstein cows were assessed using a combined arena test [consisting of exposure to consecutive novel environment (NE), novel object (NO), and novel human (NH) tests]. Personality traits were established by principal component analysis (PCA) of behaviors expressed in these tests. The PCA of cow behaviors during the precalving test revealed 3 factors, or personality traits, interpreted as exploratory, active, and bold. The PCA of cow behaviors during the postcalving test revealed 2 factors, interpreted as active and exploratory. From these cows, 23 female Holstein heifers were produced and enrolled in this study at 7 mo of age. Heifers were personality tested once through a similar combined arena test. The PCA of heifer behaviors during the NO test revealed 3 factors, interpreted as bold, exploratory-active, and social. The PCA of heifer behaviors during the NH test revealed 2 factors, interpreted as exploratory-active and social. All factor scores from each cow and heifer pair were tested for association. An association between cows with higher scores on the factor interpreted as exploratory and heifers with lower scores on the factor interpreted as bold was detected. There were tendencies for cows with higher scores on the factor interpreted as active to be associated with heifers that scored highly on the factors interpreted as exploratory-active and bold. The data suggests that there are some limited associations between personality traits of cows and their heifer offspring; further exploration of these associations may lead to the prediction of heifer personality based on cow personality.

## Highlights

• Personality traits identified in cows were associated with those in their heifers.
• Some personality traits were similar in cows and their heifers, but some were not.
• The results add to the literature exploring heritability of personality in cattle.
• There may be potential for considering personality traits when breeding cattle.

## Graphical Abstract

**Figure:**
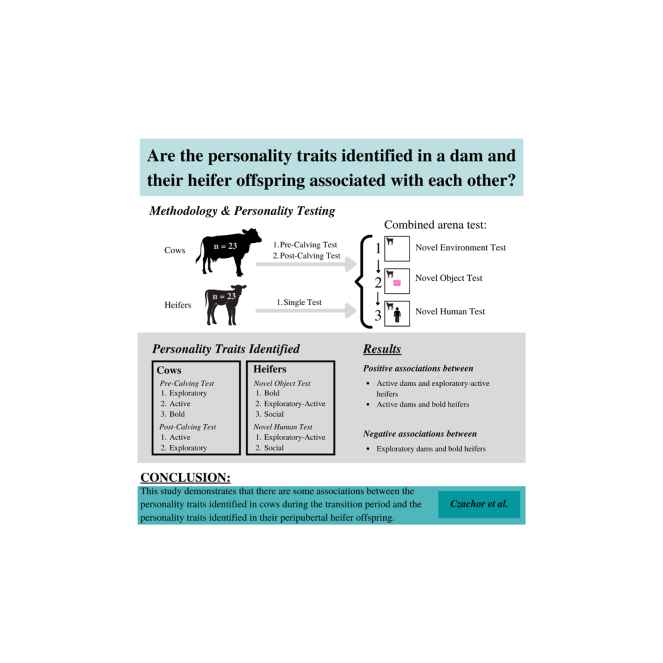
**Summary:** This study explored potential associations between the personality traits identified in cows during the transition phase and the personality traits identified in their peripubertal heifer calves. The 23 cows and heifers studied were personality tested using a combined arena test consisting of 3 consecutive stages: a novel environment, novel object, and novel human test. Personality traits were established by principal component analyses of behaviors expressed during the stages of the combined arena test. There were linear associations between the personality trait scores identified in the cows and their heifer offspring. The results demonstrate that some personality traits were similar in the cows and heifers studied, including positive relationships between active cows and exploratory-active heifers and active cows and bold heifers. Alternatively, there was a negative association between exploratory cows and bold daughters. Further exploration of these associations may lead to the prediction of heifer personality based on dam personality.

Animal personality is defined as a set of individual behavioral traits that are correlated and consistent over time and across contexts and is often described through 5 factors: exploration, activity, aggressiveness, sociability, and boldness (Finkemeier et al., 2018). Individual animal personality traits influence the behavioral variation displayed within a group of animals and can help explain differences in welfare and production performance, but are not often included in breeding indexes for dairy cattle (Haskell et al., 2014). For example, temperament, a genetic underlying factor influencing personality (Finkemeier et al., 2018), is negatively correlated with ADG in beef cattle (Hoppe et al., 2010; Haskell et al., 2014). Furthermore, Llonch et al. (2018) demonstrated that dominant beef steers spent more time feeding, while more temperamental or excitable ones had higher activity levels and ate less DM. There is less work on dairy cattle; however, milking temperament is already often selected for as it is positively correlated with higher yields, faster milking speed, and herd longevity (Adamczyk et al., 2013; Haskell at al., 2014).

Some temperamental and personality traits have been reported to have a large range in heritability values (Haskell et al., 2014; Koolhaas and Van Reenen, 2016; Finkemeier et al., 2018). For example, reported heritability values of docility (measured by the response to a human approaching and trying to move an animal to a specific corner of the pen when separated from the herd) range from 0.00 to 0.61, and milking temperament (measured categorically by the level of response over the entire milking procedure) average between 0.07 and 0.53 (Haskell et al., 2014). Similarly, the heritability of temperament traits quantified from chute test and flight-speed test scores in beef cattle vary from 0.03 to 0.67, and 0.05 to 0.70, respectively (Hoppe et al., 2010; Haskell et al., 2014). These heritability values are similar to those of some traits currently used for genetic improvements in production, such as milk yield, which has an estimated heritability of 0.24 (Visscher and Goddard, 1995; Haskell et al., 2014).

Overall, it is suggested that personality traits are definable and quantifiable, heritable at an adequate level, and can positively correlate to production traits (Haskell et al., 2014). However, more research needs to be done addressing dairy cattle personality to consider selection opportunities, especially what level of traits are passed from dam to calf (Haskell et al., 2014; Koolhaas and Van Reenen, 2016). Thus, the objective of this study was to explore potential associations between personality traits identified in cows by a combined arena test consisting of 3 consecutive conventional personality tests [novel environment/arena (**NE**), novel object (**NO**), and novel human (**NH**)] during the transition phase and the personality traits, identified by a similar combined arena test with the same stages, in their peripubertal heifer calves. It was hypothesized that heifer personality traits would be positively correlated to that of their dam.

All animal use and experimental procedures complied with the Canadian Council on Animal Care (2009) guidelines and were approved by the University of Guelph Animal Care Committee (protocol #4109). The dams of the female offspring used in this study consisted of 23 Holstein cows (11 primiparous and 12 multiparous; 1.7 ± 0.76 lactations; mean ± SD), that calved between June and October 2021, which were part of another research trial focused on how personality traits affect adaptation to an automated milking system (**AMS**) (Brasier et al., 2023). Those cows were housed at the University of Guelph, Elora Research Station, Ontario Dairy Research Centre (Elora, ON, Canada) for the entirety of the study period. Before calving, cows were housed in multiple freestall pens. Cows were moved to individual pens for calving and ~3 d after, after which they were moved to a single free traffic AMS pen with freestalls, where primiparous and multiparous cows, alike, were all introduced to the AMS for the first time. Those cows were selected from a larger group in that other trial (n = 60), on the basis that they produced female (heifer) offspring. The Holstein heifers (n = 23) were enrolled in the study and housed in multiple freestall pens located at the Ontario Dairy Research Centre. The heifers were all group housed, exposed to the same feeding practices, and managed under the same standard operating procedure from birth to the time of testing. For inclusion in the study, heifers had to be at least 210 d of age at the planned date of personality testing. Both heifers and cows had to be free from any known health concerns and were not tested on a day where they were suspected or confirmed to be in heat by physical observation or the records of barn staff.

Cows were personality tested twice (24 ± 3 d before parturition and 24 ± 3 d after being introduced for the first time to the AMS) using a combined arena test (as originally described by Schwanke et al., 2022, and adapted by Brasier et al., 2023). Two tests were done to address an objective of the larger trial these cows were selected from, which was to observe whether the behaviors demonstrated during the personality tests remained consistent from before parturition to afterward (Brasier et al., 2023). Testing was done on each animal individually between 1100 h and 1300 h, following the same procedure. In short, the novel observational arena was 5.8 × 10.7 m (length × width) and cows were socially isolated with limited visual contact with conspecifics. The same observational arena was used for the precalving and postcalving tests. The arena floor consisted of rubber mats and the perimeter was reinforced by a concrete wall, a concrete wall topped with metal bars, and 2 walls of metal fencing. The arena center was marked with chalk, as were 4 quadrants and 2 squares outlining 1 m and 2 m from the center. The test consisted of 3 consecutive stages: a 10-min NE test; a 10-min NO test, using a 44.5 × 49 × 76 cm chair precalving and 60.5 × 45 × 39.5 cm pink bin postcalving; and a 10-min NH test, where both the human and clothing worn (bright blue coveralls with reflective banding) were novel. During the NE stage, the cow was left alone for 10 min. The NO stage began immediately after, when the novel object was placed in the center of the arena by the familiar researcher and the cow was left alone with it for 10 min, after which it was removed. Immediately afterward was the NH stage, when a novel human walked slowly to the middle of the pen and stood still, with their back toward where they entered the pen, for 10 min. The novel human did not make direct eye contact with the cow. Each personality test was video recorded with 2 video cameras to ensure the entire pen was covered. One camera (MAX, GoPro Inc.) was attached to a metal bar just behind and above an arena wall to record all areas of the arena lengthwise. The other camera (Handycam Camcorder, Sony Corporation) was placed on a tripod of similar height behind the far corner of the adjacent wall to record the arena as a back-up.

Heifers were assessed once (age at test = 283.2 ± 34.8 d; mean ± SD, range = 232–335 d of age at test) for personality traits using a combined arena test very similar to that conducted on the cows (their dams). Animals were enrolled if at least 7 mo of age at the planned date of testing, as they were assumed to have begun the process of sexual maturity. Heifers were tested individually in a socially isolated arena, starting with the oldest animal, at a rate of 6 heifers per week. The testing arena was novel to the animals: part of an alley in the center of the barn adjacent to the pens, mainly used for staff movement, tractor access, and moving precalving age heifers. The arena measured 7.70 × 3.35 m (length × width) with concrete flooring. The walls were primarily concrete topped with metal fencing and gates, with one side having opaque white plastic sheets covering the fencing. The gates of the adjacent pens were covered temporarily by opaque tarps to limit visual contact with conspecifics. The center of the arena was marked with pink, fluorescent duct tape, as well as the edges of the 1-m radius surrounding the center. The pink tape was also used to mark the fencing and gates to divide the pen into 4 equal quadrants. All tests were conducted between 1200 h and 1700 h, and movement and noise of farm staff and researchers were limited during testing. Before testing, a familiar researcher calmly led the heifer from their home pen to the arena. The test began when the heifer had all 4 hooves in the arena. The personality test consisted of 3 consecutive stages: a 10-min NE test; a 5-min NO test, using the same 60.5 × 45 × 39.5 cm pink bin as in the cow postcalving tests; and a 5-min NH test, where both the human and clothing worn (same coveralls as those worn for the cow tests) were novel. The transitions between these stages and the way the NH conducted themselves during testing were the same as with the cows. The lengths of these tests were chosen after review of the work by researchers conducting similar tests (Kilgour, 1975) and considering efficiency in the time it would take to test all the animals in the group.

To record the combined arena test for each heifer, 2 video cameras were used so that all parts of the arena would be visible during analysis. The main camera (MAX, GoPro Inc.) was set up ~3 m above the arena to record all areas of the pen, and the second camera (HERO, GoPro Inc.) was attached to the metal fencing facing toward the middle of the pen from one side, to more accurately capture proximity to the NO and NH.

Personality traits were assessed by analyzing the 30-min (cow) and 20-min (heifer) recordings for the frequencies, durations, and latencies of specified behaviors during each stage of the combined area test (NE, NO, and NH). The ethogram used for the cow tests is reported in Brasier et al. (2023) and was adapted from that previously presented by Schwanke et al. (2022). See Table 1 for the ethogram used for the heifer tests. Behaviors were chosen based on previous studies (Gibbons et al., 2009; Neave et al., 2018) and the ethogram of Schwanke et al. (2022) to record those that pertain to the 5 commonly described personality factors in animal research (Finkemeier et al., 2018). Videos of the cow tests were analyzed continuously by a single researcher after being trained through a detailed explanation of the ethogram and confirming intraobserver and interobserver reliability. Video analysis of the heifer tests was also done continuously and performed by a single researcher with Solomon Coder, after establishing interobserver reliability of κ > 0.68 for each test between that researcher and another person. All variables representing a duration of time were converted to a % of the total time for that stage of the combined arena test (i.e., time spent performing the behavior divided by the time allotted to the respective stage of the test).

**Table 1:** Definitions of behavioral measures and events recorded during the combined arena test for assessment of personality traits of heifers (n = 23)

Behavior	Definition
Recorded during novel arena/environment, novel object, and novel human tests	
Quadrant change^1^ (count)	Total number of quadrants crossed with all hooves.
Vocalization (count)	All sounds emitted by the heifer.
Urination/defecation^2^ (count)	Heifer urinates or defecates.
Exploring (s)	Time spent sniffing or licking walls or ground while moving or standing still. Muzzle or tongue must be ≤15 cm from the wall or ground.
Inactive (s)	Time spent standing still (all 4 hooves for ≥2 s) without sniffing or licking walls or the ground.
Vigilance^3^ (s)	Time spent with muzzle raised above the pen gates in attempt to observe the surrounding environment.
Recorded during novel object and novel human tests only	
Avoidance distance^2^ (m)	Smallest distance between the heifer and the novel object or human during the test (>1 m, ≤1 m, or 0 m/contact; measured by proximity of both front hooves to arena markings).
Touching novel object/human (s)	Time spent sniffing or licking the object. Muzzle or tongue must be ≤5 cm from the object.
Latency to contact novel object/human (s)	Time from start of test to first touch (≤5 cm) of the novel object or human.
Attentive (s)	Time spent with head oriented toward the object or human. Heifer cannot be touching or have their front hooves ≤1 m from the object or human.
Nearby (s)	Time spent with front hooves ≤1 m from the object or human but not touching them or behind the novel human.
Behind human^2^ (s)	Time spent with front hooves ≤1 m behind the novel human but not touching it.

1 Non-normally distributed, square-root transformed data used in later analysis.

2 Not used in principal component analysis as behaviors occurred infrequently and did not meet assumptions of normality.

3 Non-normally distributed, log_10_ transformed data used in later analysis.

Statistical analyses were conducted using SAS (version 9.4; SAS Institute Inc.) utilizing data from the observed cows (n = 23 experimental units) and heifers (n = 23 experimental units). Significance was declared if *P* ≤ 0.05 and tendencies were considered at 0.05 < *P* ≤ 0.10. All data were screened for normality using the UNIVARIATE procedure of SAS; heifer quadrant changes and vigilance were square-root and log_10_ transformed, respectively, to meet assumptions of normality. Transformations of cow data are reported by Brasier et al. (2023). Behaviors scored from different test stages within the combined arena test (NE, NO, and NH) were then analyzed for association using the CORR procedure. If the behaviors were significantly associated (defined at *P* < 0.05), then the value was averaged across all the test stages that measured that specific behavior. For the cow tests, the behaviors averaged were vocalization, quadrant change, walking, vigilance, escape, exploring (precalving test only), time in quadrant 1 (precalving test only), time in quadrant 3, contact (precalving test only), and time <1 m from the NO and NH (precalving test only). No heifer behaviors were averaged.

Separate principal component analyses (**PCA**) were performed for the cow and heifer tests in SAS using the FACTOR procedure with varimax rotation. Personality traits were identified by condensing correlated behavioral measures from the combined arena test. For cows, behaviors from the NE, NO, and NH tests were analyzed in 2 separate PCA, representing the precalving and postcalving tests (sampling adequacy of 0.73 and 0.60, respectively), as described by Brasier et al. (2023). The behaviors that had communality estimates ≥0.30 were used in each respective PCA. These were quadrant change, latency to contact the NH, locomotion, escape, exploring, contact with the NO and NH, time spent within 1 m of the NO and NH, time spent greater than 2 m from the NO and NH, and the time spent behind the NH (precalving); and quadrant change, latency to contact the NH, locomotion, escape, exploring in the NH test, and time spent in contact with the NH (postcalving) (Brasier et al., 2023). Examination of scree plots led to retaining factors with eigenvalues above 1.0. This resulted in 3 factors from the precalving test PCA (75.5% cumulative variance) and 2 factors from the postcalving test PCA (78.8% cumulative variance).

For the heifers, behaviors from the 3 test stages had to be analyzed in separate PCA to achieve appropriate sampling adequacy (≥0.50); only the NO and NH tests met this criterion (0.67 and 0.58, respectively) so these were analyzed further. Behaviors included in the NO PCA were quadrant change, vocalization, inactive, exploring, vigilance, attentive, touch duration, duration nearby, and the latency to contact. In the NH PCA, those behaviors were quadrant change, vocalization, inactive, exploring, vigilance, and attentive. All other variables were removed due to low communality estimates (<0.30). Examination of scree plots led to retaining factors with eigenvalues above 1.0. This resulted in 3 factors from the NO test PCA (81.2% cumulative variance) and 2 factors from the NH test PCA (73.8% cumulative variance). Behaviors from the tests for both the cows and heifers were considered high loadings if equal to or greater than ±0.63 on each factor. Individual cow and heifer scores for each respective factor (interpreted as a personality trait) were extracted using the regression procedure, indicating each animal's place on a linear scale ranging from highly negative to highly positive. To verify the stability of the heifer PCA, heifers were classified into 2 groups based on age at the time of personality testing (group 1: >290 d old, n = 11; group 2: <290 d old, n = 12), and the residuals of each test behavior were obtained from a generalized linear model with age group as the fixed effect. The PCA was then repeated using the residuals from each linear model as input variables. The results produced a similar factor loading pattern as the PCA on original observations, verifying that the observed factors (reported herein) could be interpreted as putative personality traits.

To assess our primary objective of exploring the association of the personality traits of the heifers to those of their respective dam, all scores for factors retained in the multiple PCA for the cows and their corresponding heifers were plotted against each other as paired observations. Following visual assessment, linear regression models were built using the REGRESSION procedure of SAS to assess possible associations between factor scores of the heifers and their dams; associations with *P* ≤ 0.1 are described.

The complete cow PCA data are reported by Brasier et al. (2023). In brief, in the precalving PCA, cows who scored highly on factor 1 were interpreted as being “exploratory” (high positive loadings for exploring as well as time spent in contact with and 1 m from the NO and NH, and a highly negative loading for latency to contact the NH), those who scored highly on factor 2 were interpreted as being “active” (high positive loadings for quadrant changes, locomotion, and escape behavior), and those who scored highly on factor 3 were interpreted as being “bold” (highly negative loadings for time spent behind the NH and ≥2 m from the NO and NH). In the postcalving PCA, cows who scored highly on factor 1 were interpreted as being “active” (high positive loadings for quadrant changes, locomotion, and escape behavior) and those who scored highly on factor 2 were interpreted as being “exploratory” (high positive loadings for exploring and time spent in contact with the NH, and a highly negative loading for latency to contact the NH). Further, within cow, there was a moderate positive association between the factor interpreted as “active” and a weak positive association between the factor interpreted as “exploratory” when comparing factor scores from the precalving and postcalving tests (Brasier et al., 2023).

Two PCA were also performed to identify the personality traits of the heifers, representing behavioral data from the NO and NH stages of the combined arena test separately (Table 2). In the NO PCA, those who scored highly on factor 1 were interpreted as being “bold” (high positive loadings for the time spent in contact with the NO and nearby, and a highly negative loading for the latency to contact the NO). Heifers who scored highly on factor 2 were interpreted as being “exploratory-active” (a high positive loading for exploring and highly negative loadings for inactive and attentive). Last, heifers who scored highly on factor 3 were interpreted as being “social” (high positive loadings for vigilance, quadrant changes, and vocalizations). In the NH PCA, heifers who scored highly on factor 1 were interpreted as being “exploratory-active” (a high positive loading for exploring and highly negative loadings for inactive and attentive to the NH). Heifers who scored highly on factor 2 were interpreted as being “social” (high positive loadings for vigilance, quadrant changes, and vocalizations).

**Table 2:** Coefficients (loadings) of the eigenvectors for the factors extracted from each principal component analysis (PCA) of behaviors recorded when heifers (n = 23) were subjected to a combined arena test^1^, ^2^

Test	Behavior	Factor 1	Factor 2	Factor 3
Novel object test				
	Quadrant changes^3^	0.34	0.16	**0.81**
	Vocalization	0.09	−0.01	**0.83**
	Inactive	−0.22	**−0.90**	−0.27
	Exploring	−0.12	**0.97**	−0.07
	Vigilance^4^	0.10	0.21	**0.84**
	Attentive	−0.47	**−0.69**	−0.29
	Duration of interaction	**0.87**	0.10	0.19
	Duration spent nearby	**0.87**	0.01	0.05
	Latency to contact	**−0.84**	−0.15	−0.25
Eigenvalues		4.13	1.78	1.39
Variance explained (%)		45.9	19.8	15.5
Interpretation^5^		Bold	Exploratory-active	Social
Novel human test				
	Quadrant changes^3^	0.23	**0.84**	**—**
	Vocalization	0.17	**0.80**	**—**
	Inactive	**−0.84**	−0.43	**—**
	Exploring	**0.91**	0.09	**—**
	Vigilance^4^	0.01	**0.77**	**—**
	Attentive	**−0.81**	−0.04	**—**
Eigenvalues		3.02	1.40	**—**
Variance explained (%)		50.4	23.4	**—**
Interpretation^5^		Exploratory-active	Social	**—**

1 Combined arena test consisted of successive exposure to a novel arena (10 min), novel object (5 min), and novel human (5 min). In the first PCA, the first 3 factors were extracted from behaviors observed during the novel object stage, and in the second PCA, the first 2 factors were extracted from behaviors observed during the novel human stage. Behaviors observed during the novel arena stage had too low sampling adequacy to include in further analysis.

2 High loadings (≥|0.63|) are shown in bold, indicating behavior variables that were highly correlated within each factor.

3 Variable was square-root transformed to meet assumption of normality.

4 Variable was log_10_ transformed to meet assumption of normality.

5 Personality traits were interpreted based on behaviors exhibited during the combined arena test that loaded highly (either positively or negatively) onto each factor.

The traits interpreted from the heifer and cow PCA are highly consistent with the most commonly defined personality traits (Finkemeier et al., 2018). Neave et al. (2020) compared personality traits of dairy cattle over time using behavioral responses to a similar set of 3 personality tests as used in our study. Those researchers reported 2 PCA components, interpreted as personality traits reflecting “boldness” (high positive loadings for time spent touching and playing with a NO or NH, and highly negative loadings for attentiveness and latency to touch a NO or NH) and “exploration-activity” (high positive loadings for either exploratory or active behavior, including highly negative loadings for inactivity), which showed consistency from puberty to lactation (Neave et al., 2020). The heifers in the current study that scored highly for the factors interpreted as “bold” and “exploratory-active” demonstrated similar behaviors to these in the combined arena test and were used as part of the PCA, contributing to the interpreted trait (bold: a high positive loading for the duration of interaction with the NO or NH and a highly negative loading for the latency to contact the NO or NH; exploratory-active: a high positive loading for exploring and highly negative loading for inactivity). Furthermore, the behaviors loading highly on the retained factors for both the heifer and cow PCA in our study show good discriminant validity, further strengthening the interpretations of these factors as personality traits. This is important as the behaviors expressed in personality tests are indicative of underlying personality traits; it is thought that while exploration likely reflects a balance between curiosity or the motivation to explore and the fear of novelty (Meagher et al., 2017), sociability reflects the motivation to be near other members of the herd, and fearfulness (opposite of boldness) can reflect an individual's resilience when faced with emotional challenges (Lecorps et al., 2018).

There tended to be a positive linear association between cows who were more “active” precalving and heifers that were more “exploratory-active” during the NO test (*P* = 0.055; R^2^ = 0.16; Figure 1A). There was a negative linear association between cows who were more “exploratory” in the precalving personality test and heifers that were more “bold” in the NO test (*P* = 0.01; R^2^ = 0.26; Figure 1B). Last, there was a tendency for a positive linear association between cows who were more “active” in the postcalving personality test and heifers that were more “bold” in the NO test (*P* = 0.07; R^2^ = 0.15; Figure 1C). No other associations between identified personality traits of the cows and heifers were detected (*P* > 0.1).

**Figure 1:**
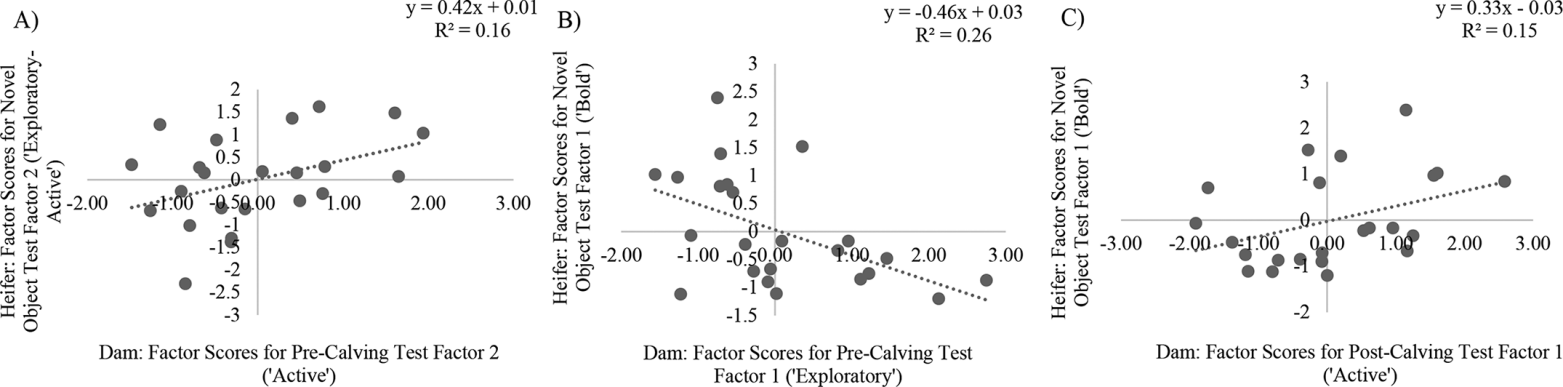
Associations between cow (n = 23) and heifer (n = 23) factor scores derived from principal component analyses (PCA) of the behaviors expressed during a combined arena test, where the retained factors are interpreted as personality traits. (A) Active PCA trait (dam; precalving test) and exploratory-active PCA trait (heifer; novel object test), (B) exploratory PCA trait (dam; precalving test) and bold PCA trait (heifer; novel object test), and (C) active PCA trait (dam; postcalving test) and bold PCA trait (heifer; novel object test).

This study contributes to a very limited body of literature where possible associations between the personality traits of dairy cows and their offspring have been explored. The results are somewhat consistent with our hypothesis that at the time of testing, heifers would have personality traits correlated to that of their dam. It was surprising that a negative linear association was detected between cow factor 1 in the precalving PCA (“exploratory”) and heifer factor 1 in the NO PCA (“bold”), since the behaviors loading highly on each respective PCA were very similar apart from the time spent exploring; they both included highly positive loadings for the duration of interaction/contact and duration of time spent near (within 1 m) the NO or NH, along with a highly negative loading for the latency to contact the NO (heifers) or NH (cows). However, the positive association between cow factor 2 in the precalving PCA (“active”) and heifer factor 2 in the NO PCA (“exploratory-active”) may suggest that the “active” personality trait and its associated behaviors are more heritable than the other identified traits. Furthermore, the “active” trait had a stronger association than that of “exploratory” when comparing the cow precalving and postcalving test factor scores (Brasier et al., 2023), suggesting that it may be a less plastic trait than others. To our knowledge, there is very limited research on this topic. Hearnshaw and Morris (1984) reported correlations of beef cow and 8-mo-old calf temperament scores, using a scale of 0 to 5 that assigned lower values to quieter animals, to be weak, with an average of 0.18. However, work in other species, such as that by Boissy et al. (2005) who performed a genetic analysis of emotional reactivity to 3 behavioral tests (combined exposure to social separation, novelty, and human contact) in sheep, have reported higher estimates of heritability. Those authors found moderate to high estimates of heritability for the behavioral traits measured during the tests, ranging from 0.14 to 0.48, providing evidence to support the possibility of considering emotional traits in selection decisions (Boissy et al., 2005). The strength of our study's correlations were to be expected, as we assumed there would be other factors affecting heifer personality traits at the age of testing. Although rearing and facility management could be controlled for, the genetic effects of sire personality traits could not (Adamczyk et al., 2013). In addition, the individual's stage of puberty reached at testing may have influenced the reliability of the heifers' personality traits, as they may be unstable during sexual maturation (Neave et al., 2020). Our assumption that the heifers studied had begun puberty may not have been correct for all individuals and may be a limitation of our study. Therefore, further work is encouraged to measure the consistency in personality traits of heifers between 7 mo of age and postbreeding, and determine the age at which they best correlate to that of their dam. Another limitation to our study could include that the cows and heifers studied were not tested in the same novel arena and some recorded behaviors differed; however, this should not affect the personality traits identified as they should be, by definition, consistent across contexts (Finkemeier et al., 2018) and be able to be identified from a variety of behaviors using the statistical methods applied. Furthermore, extending the time allotment for the NO and NH stages of the combined arena test for heifers to be the same as for their dams could yield different results or further strengthen the associations detected.

In conclusion, this novel study identified personality traits of peripubertal heifers and detected associations of those traits with that of their dams. Specifically, there was a negative association between exploratory cows and bold heifers, but also a tendency for active cows to have more exploratory-active or bold heifers. These results show that cow personality could be used as a tool to predict the personality of their heifer calves. However, further research is needed in this area to determine the earliest age this prediction can be made accurately and if their personality is maintained through further maturation.
